# *Aurantiacibacter poecillastricola* sp. nov., Isolated from the Marine Sponge, *Poecillastra wondoensis*, and Reclassification of *Erythrobacter alti* as *Aurantiacibacter alti* comb. nov.

**DOI:** 10.4014/jmb.2409.09010

**Published:** 2024-12-02

**Authors:** Soo-Bin Kim, Kyung Hyun Kim, Jin-Sook Park

**Affiliations:** Department of Biological Sciences and Biotechnology, Hannam University, Daejeon 34430, Republic of Korea

**Keywords:** *Auranciacibacter poecillastricola*, *Aurantiacibacter alti*, new taxa, marine sponge, *Poecillastra wondoensis*

## Abstract

A Gram-stain-negative, facultative anaerobic rods, designated as strain 219JJ12-13^T^, was isolated from a marine sponge, *Poecillastra wondoensis*, in Jeju-do, Republic of Korea. The cells displayed catalase and oxidase activity and were non-motile. Strain 219JJ12-13^T^ grew at 10–37°C (optimum, 25–30°C), pH 6.0–8.5 (optimum, pH 7.0–7.5), and in the presence of 0.5–6.0% (w/v) NaCl (optimum, 4.0–5.0%). The polar lipids comprised disphosphatidylglycerol, phosphatidylglycerol, phosphatidylcoline, phosphatidylethanolamine, sphingoglycolipid, two aminophosphoglycolipid, unidentified phospholipid, and two unidentified lipids. The isoprenoid quinone was identified as Q-10, and predominant cellular fatty acids were C_17:1_ *ω*6*c*, summed feature 3 (C_16:1_ *ω*7*c*/C_16:1_ *ω*6*c*), and summed feature 8 (C_16:1_ *ω*7*c*/C_18:1_ *ω*6*c*). The G+C content of the genomic DNA was 63.3%. The 16S rRNA gene and genome sequences-based phylogenetic analyses showed that strain 219JJ12-13^T^ formed a distinct phyletic clade within the genus *Aurantiacibacter*. Genome relatedness values, including average nucleotide identity and digital DNA-DNA hybridization among strain 219JJ12-13^T^ and closely related type strains, were 74.0–80.2% and 18.2–22.8%, respectively, both markedly below the thresholds for species delineation. Based on polyphasic taxonomic approach, strain 219JJ12-13^T^ represents a novel species of the genus *Aurantiacibacter*, and the name *Aurantiacibacter poecillastricola* sp. nov. is proposed. The type strain is 219JJ12-13^T^ (= KACC 23236^T^ = LMG 33060^T^). The reclassification of *Erythrobacter alti* to the genus *Aurantiacibacter* as *Aurantiacibacter alti* comb. nov. is also proposed (= KCCM 90261^T^ = NBRC 111903^T^).

## Introduction

The family *Erythrobacteraceae* is globally distributed and was recently reclassified based on phylogenomic analysis using whole-genome, resulting in the establishment of several new genera within the family [1]. As of September 2024, the genus *Aurantiacibacter* included 14 validly published speices (https://lpsn.dsmz.de/genus/aurantiacibacter).

Members of the genus *Aurantiacibacter* were reported to have discovered in diverse marine environments, including sediment [2, 3], rhizosphere mudflat [4], seawater [5, 6], and marine sponge [7]. The genus *Aurantiacibacter* members are Gram-stain-negative, catalase-positive and oxidase-variable, pleomorphic and non-spore-forming, aerobic or facultatively anaerobic, and produce carotenoid pigments but not bacteriochlorophyll a. The major ubiquinone is ubiquinone-10 (Q-10). The major fatty acid profiles and polar lipids are summed feature 8 (C_18:1_ *ω*7*c* and/or C_18:1_ *ω*6*c*) and phosphatidylethanolamine (PE), respectively. The DNA G+C content ranged between 58.3 and 67.2% [1].

The *Porifera* phylum (sponges) represent one of the earliest groups of multicellular organisms [8]. They form vital symbiotic associations with diverse prokaryotic communities, with these microbiomes playing crucial roles in processes that benefit both the microbes and the sponge, particularly by supporting host physiology [9, 10]. Given these interactions, isolating individual microorganisms and analyzing their functions is crucial to understanding the microbial community's role within the sponge host. Herein, we isolated candidate of novel species, designated strain 219JJ12-13^T^, affiliated with the genus *Aurantiacibacter* derived from a marine sponge, *Poecillastra wondoensis*, and taxonomically analyzed by a polyphasic approach.

## Materials and Methods

### Isolation and Culture Conditions

Strain 219JJ12-13^T^ was isolated from the marine sponge, *Poecillastra wondoensis*, collected off Jeju-do, Republic of Korea (33°34'12"N 126°41'58"E) in 2021. *P. wondoensis* was obtained through a self-contained underwater breathing apparatus. The sponge internal tissue was ground into homogenates using a mortar and pestle. The homogenates was serially diluted using artificial seawater (ASW; 20 g NaCl, 2.9 g MgSO_4_, 4.53 g MgCl_2_·6H_2_O, 0.64 g KCl, and 1.75 g CaCl_2_·2H_2_O per liter) and 100μl of dilution was cultured on Marine Agar 2216 (MA; BD, USA). After aerobic incubation at 25°C for 5 days, the colonies that grew on MA were purified by repeated subculturing on fresh MA plates, before being routinely cultured aerobically on MA at 28 °C for 3 days. Strain 219JJ12-13^T^ was preserved in Marine Broth 2216 (MB; BD, USA) supplemented with 20% (v/v) glycerol at −80°C. To compare phenotypic and chemotaxonomic characteristics, *Aurantiacibacter zhengii* KCTC 62389^T^, *Aurantiacibacter rhizosphaerae* KCTC 62379^T^, *Aurantiacibacter atlanticus* KCTC 42697^T^, *Aurantiacibacter marinus* KCTC 23554^T^, and *Erythrobacter alti* NBRC 111903^T^ were used as references strains obtained from the Korean Collection for Type Cultures and the NITE Biological Resource Center.

### 16S rRNA Gene-Based Phylogeny

PCR amplification of the 16S rRNA gene for strain 219JJ12-13^T^ was performed using the 27F (5'-AGA GTT TGA TCC TGG CTC AG-3') and 1492R (5'-GGT TAC CTT GTT ACG ACT T-3') universal primers. The PCR amplicon was purified by the HiGene Gel & PCR Purification System (Biofact, Republic of Korea). The purified PCR product was sequenced by Macrogen (Republic of Korea) using 27F, 518F (5'-CCA GCA GCC GCG GTA ATA CG-3'), 800R (5'-TAC CAG GGT ATC TAA TCC-3'), and 1492R primers [11]. The nearly complete sequence of 16S rRNA gene (1,412 nucleotides) was compared with those of reported type strains through the EzBioCloud (https://www.ezbiocloud.net/) [12]. The sequences of 16S rRNA gene were aligned using ClustalW integrated within BioEdit software [13]. Neighbor-joining (NJ), maximum-parsimony (MP), and maximum-likelihood (ML) trees were reconstructed using MEGA11 program [14] with bootstrap values (1,000 resampled datasets). For ML and NJ trees reconstruction, the Kimura's two-parameter model was used with complete gap deletion, while the MP tree was reconstructed using the Subtree-Pruning-Regrafting heuristic method with pairwise gap deletion.

### Analyses of Genome Features and Whole Genome-Based Phylogeny

Genomic DNA derived from cells of strain 219JJ12-13^T^ was extracted and purified by the HiGene Genomic DNA Prep Kit (Biofact, Republic of Korea) and sequence analyzed on the Illumina NovaSeq platform by DNA Link (Republic of Korea). Sequencing reads with high quality were *de novo* assembled using the SPAdes software (version 3.14.0) [15]. The completeness and contamination rates of assembled genome were assessed using CheckM (version 1.2.2) in NCBI assembly [16]. The genome sequence was deposited in GenBank and functional genes were predicted through the NCBI Prokaryotic Genomes Annotation Pipeline [17]. The G+C content of the genomic DNA was determined based on the whole genome sequence. A phylogenomic tree based on the concatenated 81 housekeeping core genes was constructed using the UBCG2 pipeline, with bootstrap values (1,000 replications) [18]. Average nucleotide identity (ANI) and digital DNA-DNA hybridization (dDDH) values were estimated using OrthoANI [19] and the web-based Genome to Genome Distance Calculator based on formula 2 [20], respectively. The protein function was determined through the BlastKOALA [21].

### Physiology

The growth range of strain 219JJ12-13^T^ was evaluated at various temperatures (4, 10, 15, 18, 25, 30, 37, 42, and 45°C) on MA and pH values (4–10 at 0.5 pH unit intervals) on MB for 3 days at 28°C. MB was prepared with pH ranges of 4.0–5.5, 6.0–8.0, and 8.5–10.0, using 50 mM acetate, phosphate, and Tris buffer systems, respectively, with pH adjusted after sterilization if needed. The NaCl tolerance was tested using modified ZoBell medium (laboratory formula; 5 g Peptone, 1 g Yeast extract, 5.94 g MgSO_4_·7H_2_O, 4.53 g MgCl_2_·6H_2_O, 0.64 g KCl, 0.01 g FePO_4_·4H_2_O, and 1.3 g CaCl_2_ per liter, pH 7.6) with varying NaCl concentrations (0, 0.5, and 1.0–10.0% at 1.0%intervals, w/v), and cell growth was evaluated aerobically incubation at 28°C for 3 days. Growth on anaerobic condition was assessed on MA at 28°C for two weeks using the GasPak Plus system (BD, USA). All physiological and biochemical tests were executed using cells grown on MA at 28°C for 3 days. Examination of cellular morphology and flagella were conducted using light microscope (Optiphot-2, Nikon, Japan) and transmission electron microscope (LIBRA 120 kV, Carl Zeiss, Germany), respectively. The Gram staining of cells was performed following the method described by Doetsch [22] and the potassium hydroxide method described previously [23]. Activity of catalase and oxidase was evaluated as described [24], by observing bubble production in 3% (v/v) hydrogen peroxide solution (Junsei, Japan) and color change with a 1% (w/v) *N*,*N*,*N*',*N*'-tetramethyl-1,4-phenylenediamine solution (Sigma-Aldrich, USA), respectively. Cell motility by flagella was tested on semi-solid MA (0.2% agar). Casein (1.0% skimmed milk, w/v), tyrosine (0.5%), and Tween 80 (1.0%) hydrolysis was tested on MA following established protocols [25]. Activities of enzyme and biochemical properties were analyzed using API kits including ZYM, 32GN, and 20NE (BioMérieux, France) following the manufacturer’s guidelines, with modifications of the inoculation media to include 2% sodium chloride. The test strips of API ZYM were evaluated after 6 h of incubation at 25°C, and the other API strips were assessed after 3 days of incubation at 28°C.

### Chemotaxonomic Analyses

Cells of strain 219JJ12-13^T^, *A. zhengii* KCTC 62389^T^, and *A. rhizosphaerae* KCTC 62379^T^ were cultured on MB under their optimal temperatures and harvested at the exponential growth phase to analyze polar lipids. Polar lipids were extracted and analyzed using thin-layer chromatography according to the procedure described by Minnikin *et al*. [26]. The following reagents were used for identification of various polar lipids: 10% ethanolic molybdophosphoric acid, ninhydrin, molybdenum blue, and *α*-naphthol reagent. The isoprenoid quinone of strain 219JJ12-13^T^ was extracted using chloroform : methanol mixture (2:1, v/v) and analyzed using HPLC (Waters 2487; Waters, USA) following previously described methods [27]. Profile of cellular fatty acids was analyzed for strain 219JJ12-13^T^, *A. zhengii* KCTC 62389^T^, *A. rhizosphaerae* KCTC 62379^T^, *A. atlanticus* KCTC 42697^T^, *A. marinus* KCTC 23554^T^, and *E. alti* NBRC 111903^T^ after cultivation on MA under their respective optimal growth conditions for 3 days. Saponification, methylation, and extraction of cellular fatty acids were performed following the standard MIDI protocol. The fatty acid methyl esters was analyzed using gas chromatography (model 6890; Hewlett Packard, USA) and identified using the TSBA6 database within the Microbial Identification System (Sherlock ver. 6.3) [28].

## Results and Discussion

### Phylogeny Based on 16S rRNA Gene Sequences

**Fig. 1:**
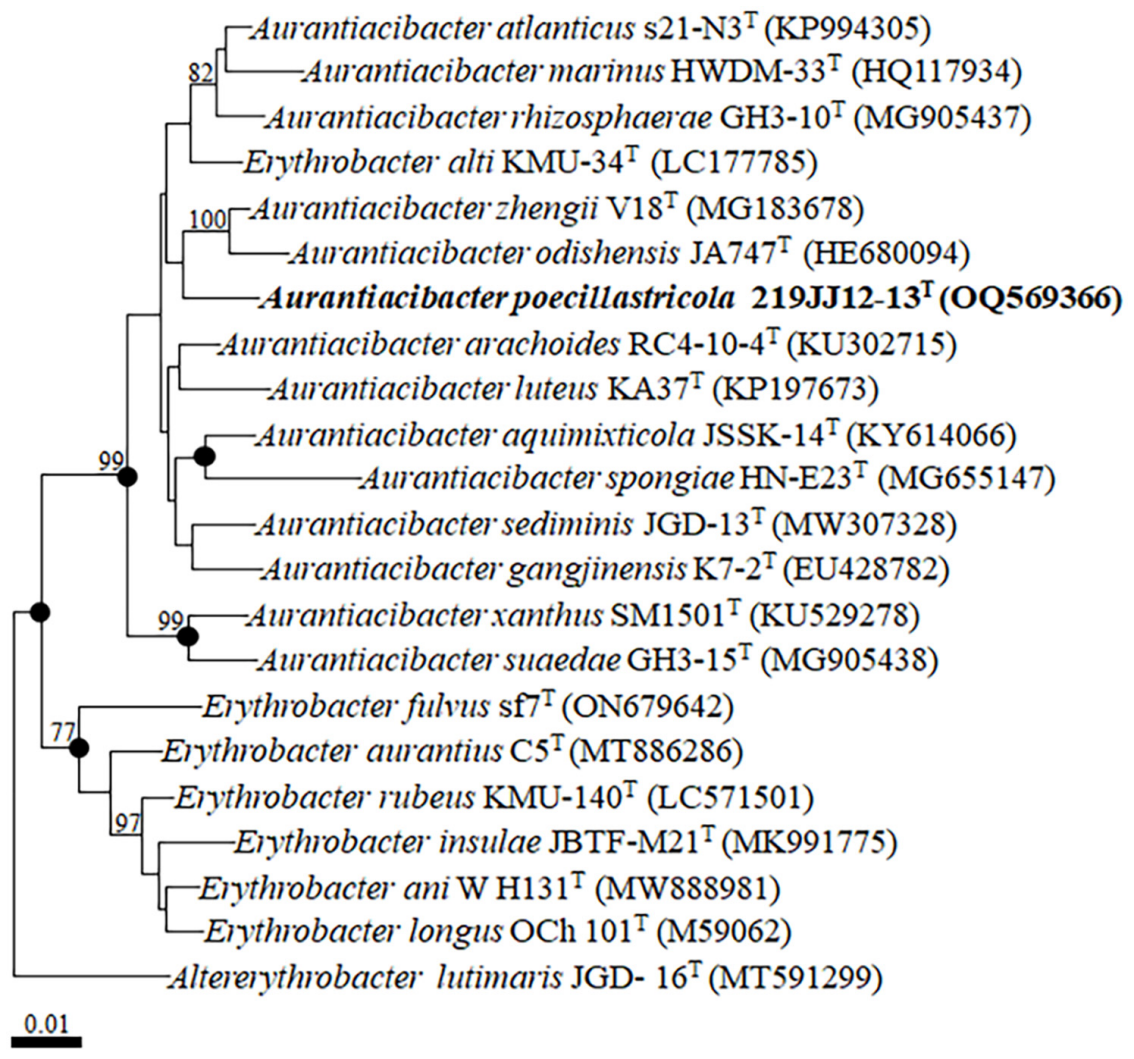
A neighbor-joining tree based on 16S rRNA gene sequences, showing the phylogenetic relationships of strain 219JJ12-13^T^ and their closely related taxa. Bootstrap values above 70% are shown on nodes in percentages of 1,000 replicates. Filled circles (●) indicate the corresponding nodes that were also recovered in the trees generated with maximum-likelihood and maximum-parsimony algorithms. Altererythrobacter lutimaris JGD-16^T^ (MT591299) was used as the outgroup. Scale bar equals 0.01 changes per nucleotide position.

Comparative analysis of 16S rRNA gene sequences revealed that strain 219JJ12-13^T^ was closely related to *A. zhengii* V18^T^ and *A. rhizosphaerae* GH3-10^T^, with sequence similarities of 98.2% and 98.1%, respectively. It also shared 97.5% similarity with *A. marinus* HWDM-33^T^, *A. atlanticus* s21-N3^T^, *A. odishensis* JA747^T^, and *Erythrobacter alti* KMU-34^T^. The NJ tree showed that strain 219JJ12-13^T^ clustered with *A. zhengii* V18^T^ and *A. odishensis* JA747^T^, forming a distinct phyletic lineage within the genus *Aurantiacibacter* (Fig. 1), which was consistent with MP tree. In ML phylogenetic tree, although overall tree topology is quite different from the others, strain 219JJ12-13^T^ also constituted a phyletic lineage (Fig. S1). Phylogenetic analyses suggested that strain 219JJ12-13^T^ may be a member of the genus *Aurantiacibacter*. Additionally, *E. alti* KMU-34^T^ was formed a phyletic lineage with members of the genus *Aurantiacibacter* in phylogenetic trees (Figs. 1 and S1), which suggested that *E. alti* may be re-classified to the genus *Aurantiacibacter*.

### General Genomic Features and Whole Genome-Based Phylogeny

**Fig. 2:**
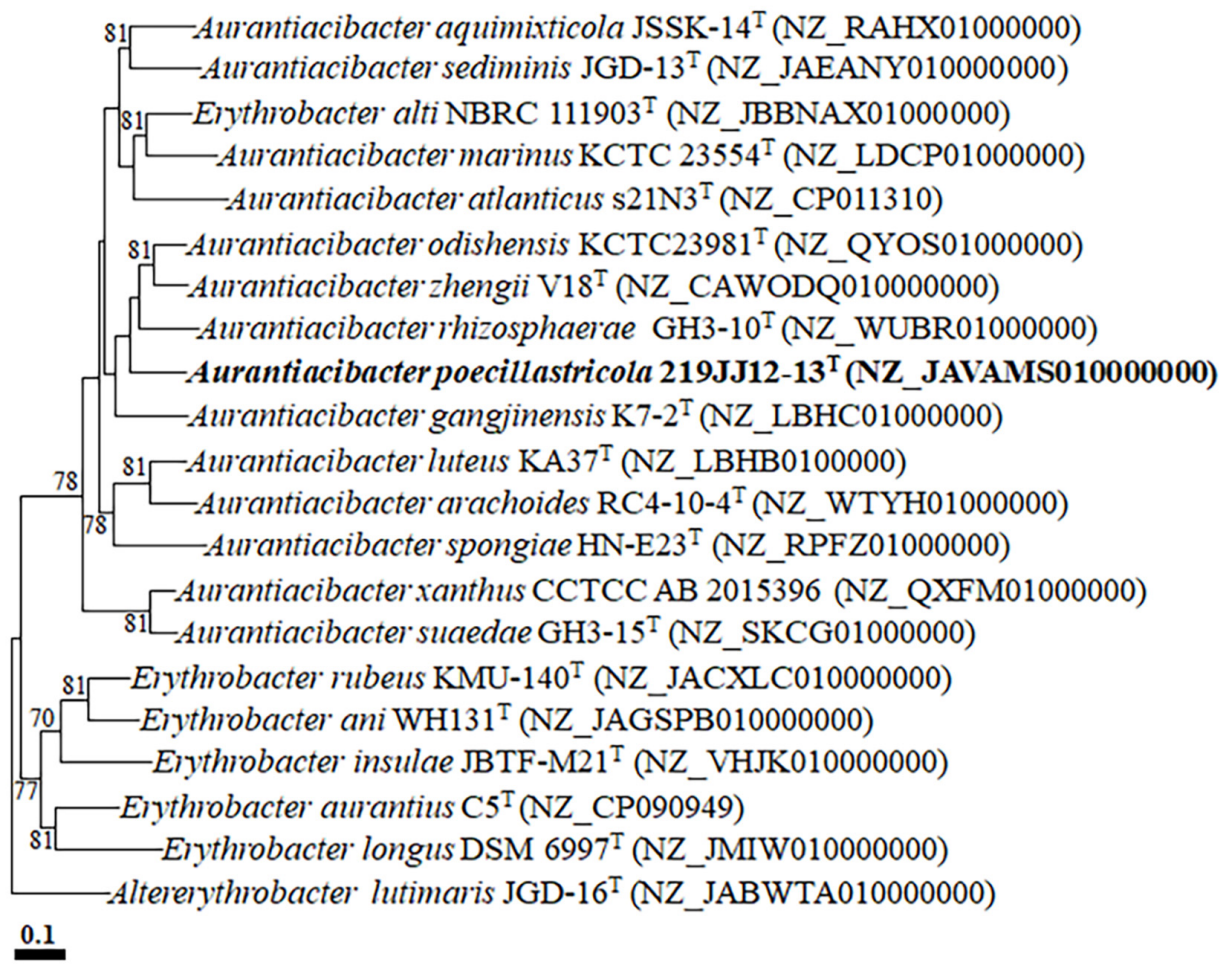
A phylogenomic tree based on concatenated nucleotide sequences of 81 housekeeping core genes showing phylogenetic relationships between strain 219JJ12-13^T^ and closely related type strains. Bootstrap values (> 70%) based on 1,000 replicates are shown on branch nodes. Altererythrobacter lutimaris JGD-16^T^ (NJ_JABWTA010000000) was used as an outgroup. The scale bar equals 0.10 changes per nucleotide position.

The genome of strain 219JJ12-13^T^ consisted of a total size of 3,881,864 bp distributed across 34 contigs (N50 value of 1,211,024 bp) and the G+C content of genomic DNA was 63.3%. The genome contained a total of 3,784 genes, including 3,733 protein-coding sequences, 3 rRNAs (one 5S rRNA, one 16S rRNA, and one 23S rRNA), and 45 tRNAs. Completeness and contamination rate of genome, as assessed by CheckM, were 99.6% and 1.0%, respectively, indicating a high-quality assembly. The sequence of 16S rRNA gene obtained through Sanger sequencing showed completely identical to the sequence derived from genome sequence. Genomic features of strain 219JJ12-13^T^ and reference strains were presented in Table S1. Phylogenomic analysis showed that strain 219JJ12-13^T^ clustered with *A. odishensis* KCTC23981^T^, *A. zhengii* v18^T^, *A. rhizosphaerae* GH3-10^T^, and *A. gangiinensis* K7-2^T^, forming a distinct phyletic lineage. Additionally, *E. alti* NBRC 111903^T^ also clustered with *A. marinus* KCTC 23554^T^ and *A. atlanticus* s21N3^T^, forming a phyletic lineage within the genus *Aurantiacibacter* (Fig. 2). The genome relatedness between strain 219JJ12-13^T^ and members of the genus *Aurantiacibacter*, based on ANI and dDDH analyses, were 74.0–80.2% and 18.2–22.8% range, respectively (Table S1), significantly lower than the thresholds (ANI value of 95–96% and dDDH value of 70%) for species delineation [29]. These analyses indicate that strain 219JJ12-13^T^ constitutes a novel species and *E. alti* can be a member of the genus *Aurantiacibacter*.

### Sponge Symbiosis-Associated Genes

Sponge symbionts, such as marine heterotrophic and photoautotrophic microorganisms, are essential for sponge-host survival. Symbiotic bacteria provide vitamin Bs (biotin, folate, and cobalamin), that the sponge cannot synthesize, thereby enhancing their fitness, and survival [30, 31]. Additionally, valine, leucine, and isoleucine, known as branched chain amino acids (BCAAs), have been reported to support muscle growth, energy production, and protein synthesis [32-35].

Bioinformatic analysis revealed genes in the genome that were involved in the synthesis of biotin (vitamin B7) from pimeloyl-CoA (*bioABDF*) and folate (vitamin B9) from GTP (*folABCEKP* and *phoA*). Additionally, strain 219JJ12-13^T^ possesses genes for the synthesis of BCAAs, including L-valine from pyruvate (*ilvBCDE*), L-leucine from pyruvate (*ilvBCDE* and *leuABC*), and L-isoleucine from threonine or pyruvate (*ilvABCDE*). The genomic potential for biosynthesizing vitamin Bs and BCAAs of strain 219JJ12-13^T^ contributes to enhancing the physiological condition of the host sponge.

### Physiology

Colonies of strain 219JJ12-13^T^, cultured on MA at 28°C for 3 days, appeared circular, convex shape, and pale-yellow. The cells of strain 219JJ12-13^T^ were Gram-staining-negative and rods without motility (0.5–0.7 μm in width and 1.0–1.4 μm in length) (Fig. S1). Strain 219JJ12-13^T^ showed anaerobic growth on MA after two weeks. The growth conditions for strain strain 219JJ12-13^T^ were as follows: a temperature range of 10–37°C (optimal 25–30°C), a pH range of 6.0–8.5 (optimal pH 7.0–7.5), and NaCl concentrations of 0.5–6.0% (w/v) (optimal 4.0–5.0%). Temperature, NaCl, and pH growth ranges of strain 219JJ12-13^T^ showed slight differences from those of reference strains and were more similar to *A. rhizosphaerae* than to *A. zhengii*. Additionally, *E. alti* showed similar temperature, NaCl, and pH growth ranges to strains, *A. atlanticus* and *A. marinus*, with the closest phylogenetic relationship to it. Tyrosine and Tween 80 were hydrolyzed, but not casein. Phenotypic properties of strain 219JJ12-13^T^ including positive enzymatic activities of oxidase, catalase, and alkaline phosphatase and negative for fermentation of glucose, production of indole, activities of lipase (C14), *α*-galactosidase, *β*-glucuronidase, *N*-acetyl-*β*-glucosaminidase, *α*-mannosidase, and *α*-fucosidase, and assimilation of phenyl-acetate, caprate, L-rhamnose, malonate, 4-hydroxy-benzoate, D-ribose, inositol, 5-ketogluconate, D-sorbitol, and glycogen were commonly observed in strain 219JJ12-13^T^ as well as in the reference strains, including *E. alti* KMU-34^T^. Phenotypic, physiological, and biochemical analyses revealed that strain 219JJ12-13^T^ exhibited distinct assimilation and enzyme activities for various compounds, enabling its differentiation from closely related *Aurantiacibacter* species. Comparisons of phenotypical, physiological, and biochemical features of strain 219JJ12-13^T^ and the closely related type strains were listed in Tables 1 and S2.

### Chemotaxonomic Characteristics

Strain 219JJ12-13^T^ contained the following major polar lipids: disphosphatidylglycerol (DPG), phosphatidylglycerol (PG), PE, sphingoglycolipid (SGL), phosphatidylcholine (PC), two aminophosphoglycolipids (APGLs), unidentified phospholipid (PL), and two unidentified lipids (Ls) (Fig. S3). Polar lipids, such as PG, PE, SGL, and APGL were shared in strain 219JJ12-13^T^ and closely related strains, *A. zhengii* KCTC 62389^T^ and *A. rhizosphaerae* KCTC 62379^T^, however, DPG and PC varies among strains. Strain 219JJ12-13^T^ uniquely harbored DPG and PC, distinguishing it from closely related species of the genus *Aurantiacibacter*. The Q-10 was determined as sole isoprenoid quinone in strain 219JJ12-13^T^, in alignment with the reference strains, including *E. alti* [6]. The predominant fatty acid (> 10% of the total fatty acids) in strain 219JJ12-13^T^ were C_17:1_ *ω*6*c* (12.7%), summed feature 3 (C_16:1_ *ω*7*c*/C_16:1_ *ω*6*c*, 17.0%), and summed feature 8 (C_16:1_ *ω*7*c*/C_18:1_ *ω*6*c*, 38.2%). The fatty acid composition of strain 219JJ12-13^T^ was comparable to that of the reference strains, including *E. alti* (Table 2). The polar lipid composition of *E. alti* shared almost all lipids with reference strains within the genus *Aurantiacibacter*, except for DPG and PL. Also, the predominant fatty acids of *E. alti* NBRC 111903^T^ consisted of summed feature 3 (24.7%) and summed feature 8 (44.8%), which were consistent with closely related reference strains. These chemotaxonomic characteristics show that strain 219JJ12-13^T^ and *E. alti* NBRC 111903^T^ are similar to the characteristics of the genus *Aurantiacibacter*, allowing for differentiation from closely related *Aurantiacibacter* species.

### Description of *Aurantiacibacter poecillastricola* sp. nov.

*Aurantiacibacter poecillastricola* (po.e.cil.las’tri.co.la. N.L. gen. masc. n. *poecillastra*, the sponge *Poecillastra*; L. masc./fem. n. *incola*, a dweller, inhabitant; N.L. gen. of masc./fem. N. *poecillastricola*, a dweller of the sponge, *Poecillastra*).

Cells are Gram stain-negative, facultative anaerobic rods, non-motile (0.5–0.7 μm in width and 1.0–1.4 μm in length), and oxidase- and catalase-positive. Colonies on the MA are circular, convex, round, and pale yellow. Growth occurs within the range of 10–37°C (optimum, 25–30°C), pH 6.0–8.5 (optimum, pH 7.0–7.5), and NaCl concentrations of 0.5–6.0% (w/v) (optimum, 4.0–5.0%). Tyrosine and Tween 80 are hydrolyzed but casein is not.

In API 20NE kit, nitrate reduction, indole production, and glucose fermentation are negative. Positive for the assimilation of D-glucose, D-mannitol, L-arabinose, D-maltose, malate, suberate, lactate, and L-serine and negative for D-mannose, *N*-acetyl-D-glucosamine, gluconate, adipate, citrate, phenyl-acetate, D-sorbitol, L-fucose, propionate, valerate, L-histidine, 2-ketogluconate, 3-hydroxy-butyrate, L-proline, acetate, 3-hydroxy-benzoate, itaconate, L-alanine, salicin, D-melibiose, caprate, L-rhamnose, malonate, 4-hydroxy-benzoate, D-ribose, inositol, 5-ketogluconate, D-sorbitol, and glycogen. Positive for enzyme activity of arginine dihydrolase, protease, urease, leucine arylamidase, valine arylamidase, esterase (C4), esterase/lipase (C8), trypsin, *α*-chymotrypsin, acid phosphatase, naphtol-AS-BI-phosphohydrolase, *β*-galactosidase (PNPG), *β*-glucosidase, and alkaline phosphatase, and negative for cystine arylamidase, *α*-glucosidase, lipase (C14), *α*-galactosidase, *β*-glucuronidase, *N*-acetyl-*β*-glucosaminidase, *α*-mannosidase, and *α*-fucosidase. The polar lipids comprise DPG, PG, PC, PE, SGL, 2APGLs, 2 unidentified PLs, and 2 unidentified Ls. The sole isoprenoid quinone is Q-10. The major cellular fatty acids are C_17:1_ *ω*6*c* (12.7%), summed feature 3 (C_16:1_ *ω*7*c*/C_16:1_ *ω*6*c*, 17.0%), and summed feature 8 (C_16:1_ *ω*7*c*/C_18:1_ *ω*6*c*, 38.2%). The G+C content of genomic DNA of strain 219JJ12-13^T^ is 63.3%.

The type strain, 219JJ12-13^T^ (= KACC 23236^T^ = LMG 33060^T^), was isolated from a marine sponge *Poecillastra wondoensis* collected from Jeju-do, Republic of Korea. The GenBank accession numbers for the 16S rRNA gene and whole genome sequences of strain 219JJ12-13^T^ are OQ569366 and JAVAMS010000000, respectively.

### Description of *Aurantiacibacter alti* comb. nov.

*Aurantiacibacter alti* (al’ti. L. gen. neut. n. alti, of the open sea).

Basonym: *Erythrobacter alti* described by Yoon (2017).

The description is as given for *Erythrobacter alti* by Yoon [6] with the following modification. The G+C content of genomic DNA is 60.5%.

The type strain is KMU-34^T^T (= KCCM 90261^T^ = NBRC 111903^T^).

### Emended description of *Aurantiacibacter zhengii* Fang *et al*. 2019

The description is essentially in agreement with that given above for the species *Aurantiacibacter zhengii* by Fang [2], with the following modifications. The polar lipid profile includes PG, PE, PL, SGL, 2 APGLs, and 2 Ls, but not present GL, APL, AGL, and DPG. The G+C content of genomic DNA is 62.5 %.

### Emended description of *Aurantiacibacter rhizosphaerae* Lee and Kim 2020

The description is essentially in agreement with that given above for the species *Aurantiacibacter rhizosphaerae* by Lee and Kim [4], with the following modifications. The polar lipid profile includes PG, PE, PC, PL, SGL, 2 APGLs, and 3 Ls, but GL is absent. The G+C content of genomic DNA is 61.5 %.

The 16S rRNA gene and whole genome sequences of strain 219JJ12-13^T^ have been deposited in the GenBank database under accession numbers OQ569366 and JAVAMS010000000, respectively.

Phylogenetic trees based on the ML and MP algorithms, images of transmission electron microscopy, and profiles of polar lipids are provided as supplementary information.

## Supplemental Materials

Supplementary data for this paper are available on-line only at http://jmb.or.kr.



## Figures and Tables

**Table 1:** Comparisons of phenotype characteristics between strain 219JJ12-13^T^ and the closely related type strains.

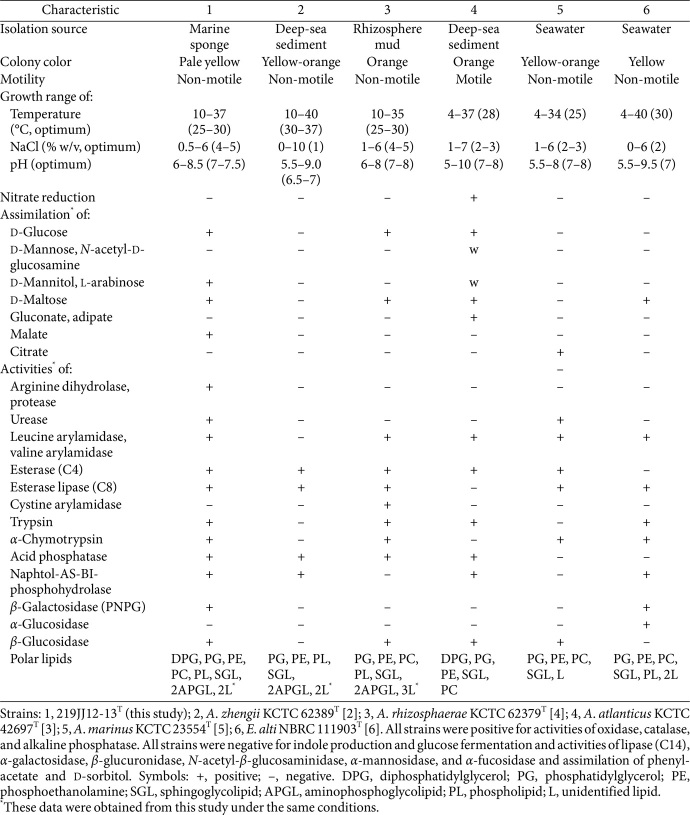

**Table 2:** Comparison of the cellular fatty acid compositions (%) of strain 219JJ12-13^T^ and the closely related type strains.

Characteristic	1	2	3	4	5	6
Saturated:						
C_12:0_	3.7	1.8	–	–	–	–
C_14:0_	1.6	1.3	1.2	–	–	–
C_16:0_	4.6	6.5	5.9	6.8	4.6	3.6
Unsaturated:						
C_17:1_ *ω*6*c*	**12.7**	8.4	5.0	3.6	6.8	2.6
Branched:						
C_18:0_ iso	–	–	–	–	4.0	2.2
C_18:1_ *ω*7*c* 11-methyl	2.7	2.7	6.8	7.7	8.3	2.2
Hydroxy:						
C_14:0_ 2OH	6.7	2.9	4.4	3.7	7.5	9.1
C_15:0_ 2OH	2.7	1.4	2.4	tr	4.1	1.3
C_16:0_ 2OH	2.8	1.5	4.8	2.2	**11.9**	2.4
C_16:1_ 2OH	1.7	–	tr	tr	1.6	1.3
C_18:1_ 2OH	–	3.0	2.9	tr	1.3	2.2
Summed feature*:						
3	**17.0**	**24.7**	**19.3**	**23.9**	7.3	**24.7**
8	**38.2**	**43.9**	**43.4**	**45.7**	**37.5**	**44.8**

Strains: 1, 219JJ12-13^T^; 2, *A. zhengii* KCTC 62389^T^; 3, *A. rhizosphaerae* KCTC 62379^T^; 4, *A. atlanticus* KCTC 42697^T^; 5, *A. marinus* KCTC 23554^T^; 6, *E. alti* NBRC 111903^T^. All fatty acid data were obtained from this study. Data are expressed as percentages of the total fatty acids, and fatty acids amounting to less than 0.5% in all strains are not shown. Major components (>10.0%) are highlighted in bold. tr, trace amount (< 1.0%); –, not detected.

*Summed features are fatty acids that cannot be resolved reliably from another fatty acid using the chosen chromatographic conditions. The MIDI system groups these fatty acids into one feature with a single percentage of the total. Summed feature 3, C_16:1_ *ω*7*c*/C_16:1_ *ω*6*c*; summed feature 8, C_18:1_ *ω*7*c*/C_18:1_ *ω*6*c*.
