# Supplementary material for: Aurantiacibacter poecillastricola sp. nov., Isolated from the Marine Sponge, Poecillastra wondoensis, and Reclassification of Erythrobacter alti as Aurantiacibacter alti comb. nov
Source: J Microbiol Biotechnol. 2024 Dec 2;35:e2409010. doi: 10.4014/jmb.2409.09010 (PMC11813356; doi:10.4014/jmb.2409.09010)
Supplement: Supplementary file 1 [file jmb-35-e2409010-supple.pdf]

Supporting Information

Fig. S1. Maximum-likelihood (A) and maximum parsimony (B) trees based on 16S rRNA gene sequences, showing the phylogenetic relationships between strain 219JJ12-13^T and closely related taxa. Bootstrap values above 70 % are shown on nodes in percentages of 1,000 replicates. *Altererythrobacter lutimaris* JGD- 16^T (MT591299) was used as the outgroup. Scale bars indicate changes per nucleotide position (A) and nucleotide changes over the whole sequence (B).

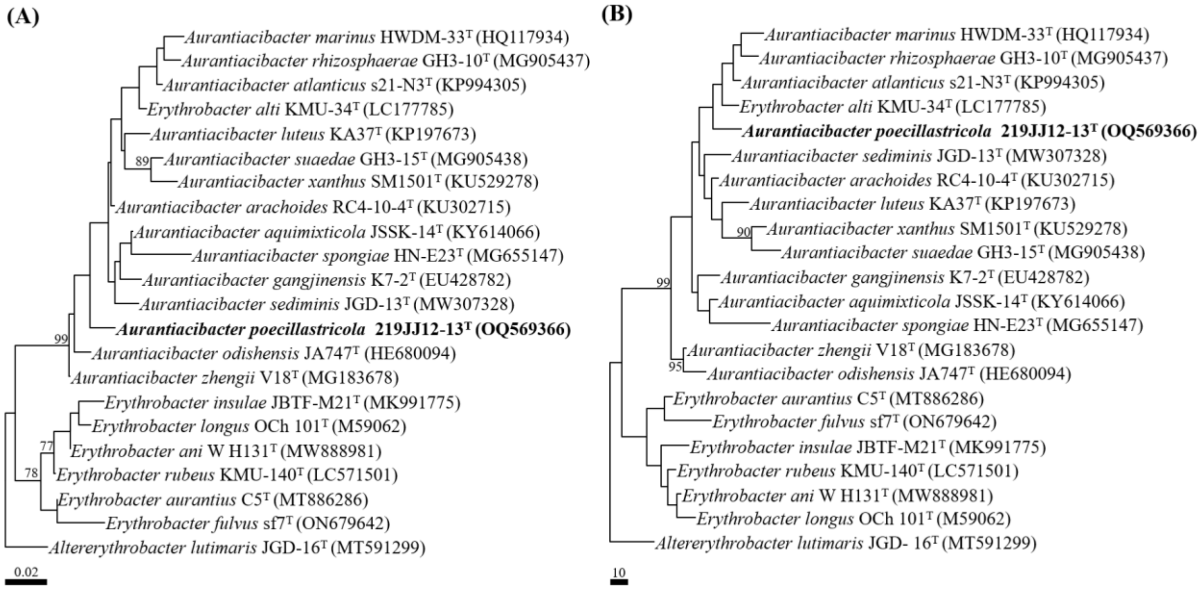

Fig. S2. Transmission electron micrographs showing the general morphology of negatively stained cells of strain 219JJ12-13^T grown on MA at 28 °C for 3 days. Bar, 1 μm.

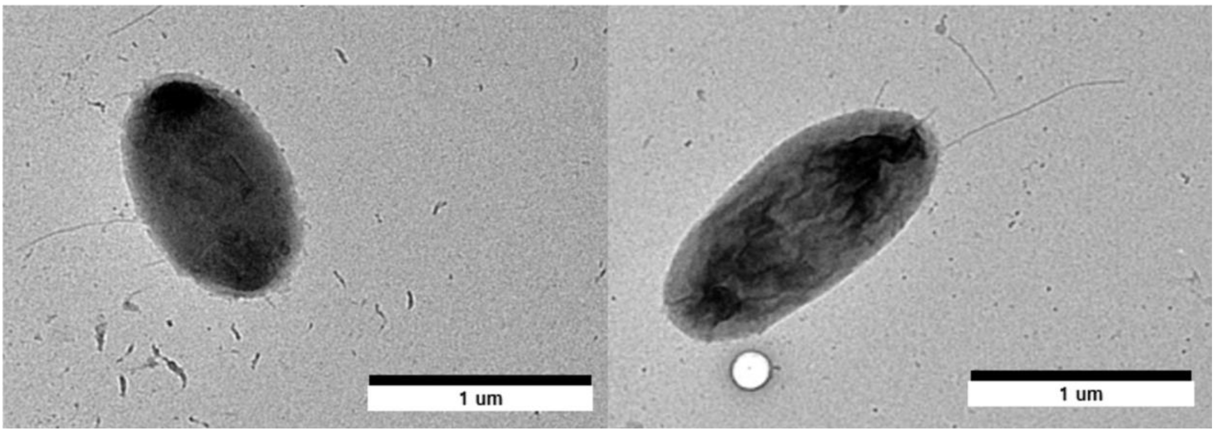

Fig. S3. Two-dimensional thin-layer chromatogram of total polar lipids of strain 219JJ12-13^{T} and two reference strains. Solvent systems: (I) chloroform-methanol-water (65:25:4, v/v/v); (II) chloroform-acetic acid-methanol-water (80:15:12:4, v/v/v/v). The TLC plates were sprayed with 10 % ethanolic molybdatophosphoric acid (A), (E), and (I), ninhydrin (B), (F), and (J), molybdenum blue (C), (G), and (K), and α-naphthol (D), (H), and (L) for the detection of total polar lipids, aminolipids, phospholipids, and glycolipids, respectively.

Strains: A–D, 219JJ12-13^{T}; E–H, *A. zhengii* KCTC 62389^{T} and I–L, *A. rhizosphaerae* KCTC 6237.

DPG, diphosphatidylglycerol; PG, phosphatidylglycerol; PC, phosphocoline; PE, phosphoethanolamine; SGL, sphingoglycolipid; APGL, aminophosphoglycolipid; PL, phospholipid; L, unidentified lipid.

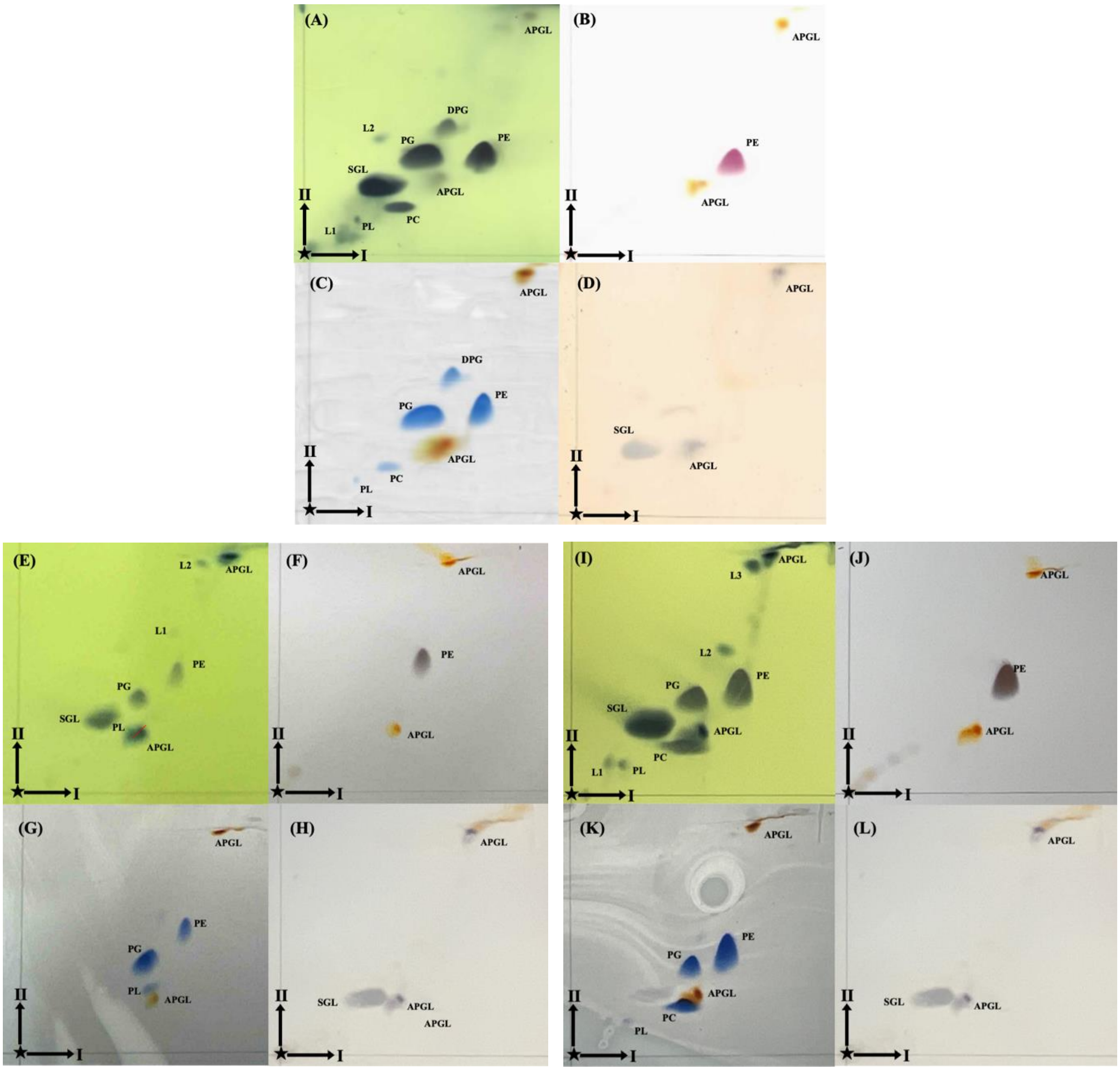

Table S1. General genomic features* of strain 219JJ12-13^{T} and the most closely related type strains.

Feature		1	2	3	4	5	6
Genome size (Mb)		3.9	3.8	3.4	3.2	2.8	2.9
Genome status		Draft	Draft	Draft	Complete	Draft	Draft
No. of contigs		37	29	6	2	5	11
N50 value (kb)		1,211.0	509.2	997.2	3,000.0	750.7	411.4
G+C content (%)†		63.3	62.5	61.5	58.5	59.0	60.5
No. of genes		3,784	3,711	3,272	3,140	2,752	2,788
No. of protein-coding genes		3,733	3,659	3,202	3,057	2,700	2,728
No. of pseudogenes		22	61	19	31	3	10
No. of tRNA genes		45	46	45	45	43	44
GenBank accession no.		JAVAMS01 0000000	QXFL01 000000	WUBR01 000000	CP011310	LDCP0100 0001	JBBNAX01 0000000
		ANI value (%)					
DDH value (%)	**1**		80.2	78.6	74.1	74.0	75.5
	**2**	22.8		79.4	74.9	74.3	75.3
	**3**	21.2	21.7		74.3	74.8	75.4
	**4**	20.9	21.3	18.6		75.2	75.9
	**5**	18.2	18.4	18.4	18.6		78.3
	**6**	19.1	19.0	18.8	19.0	20.5	

Strains: 1, 219JJ12-13^{T} (this study); 2, *A. zhengii* v18^{T}; 3, *A. rhizosphaerae* GH3-10^{T}; 4, *A. atlanticus* s21N3^{T}; 5, *A. marinus* KCTC 23554^{T}; 6, *E. alti* NBRC 111903^{T}.

*Genomic features of all strains were analyzed using the NCBI prokaryotic genome annotation pipeline (www.ncbi.nlm.nih.gov/genome/annotation_prok/).

†G+C contents were calculated from the whole genome sequence.

Table S2. Comparisons of phenotype characteristic of API 32GN between strain 219JJ12-13^{T} and the closely related type strains.

Characteristic	1	2	3	4	5	6
D-Mannitol, L-arabinose	+	–	–	*w*	–	–
D-Glucose	–	–	+	+	–	–
L-Fucose, propionate, valerate, citrate, L-histidine, 2-ketogluconate	–	–	–	–	+	–
3-Hydroxy-butyrate	–	–	+	+	+	+
L-Proline, acetate, 3-hydroxy-benzoate	–	–	–	+	+	–
N-Acetyl-D-glucosamine	–	–	–	*w*	–	–
D-Sucrose	–	–	+	–	+	+
D-Maltose	+	–	+	+	–	+
Itaconate	–	–	–	–	*w*	–
Suberate	*w*	–	–	–	–	–
Lactate, L-serine	+	–	–	–	+	–
L-Alanine	–	–	+	–	+	–

Strains; 1, 219JJ12-13^{T} (this study); 2, *A. zhengii* KCTC 62389^{T} (Fang et al. 2019); 3, *A. rhizosphaerae* KCTC 62379^{T} (Lee and Kim, 2020); 4, *A. atlanticus* KCTC 42697^{T} (Zhuang et al. 2015); 5, *A. marinus* KCTC 23554^{T} (Xu et al. 2020); 6, *E. alti* NBRC 111903^{T} (Yoon 2017). All strains were negative for assimilation of salicin, D-melibiose, caprate, L-rhamnose, malonate, 4-hydroxy-benzoate, D-ribose, inositol, 5-ketogluconate, D-sorbitol, and glycogen.
